# NTCP model guided whole brain radiation re‐planning to reduce risk of acute xerostomia and dry eye

**DOI:** 10.1002/acm2.70344

**Published:** 2025-11-18

**Authors:** Gregory Szalkowski, Jeffrey Fenoli, Mary Oakey, Xianming Tan, Kevin A. Pearlstein, Trevor J. Royce, Bhishamjit S. Chera, Shiva K. Das, Kyle Wang, Panayiotis Mavroidis

**Affiliations:** ^1^ Department of Radiation Oncology University of North Carolina at Chapel Hill North Carolina USA; ^2^ Lineberger Comprehensive Cancer Center University of North Carolina at Chapel Hill Chapel Hill North Carolina USA

**Keywords:** dry eye, LKB, NTCP, radiobiological parameters, relative seriality, whole brain radiation therapy, xerostomia

## Abstract

**Introduction:**

Previous work has shown that whole brain radiation (WBRT) can lead to acute xerostomia and dry eye from dose delivered to the parotid and lacrimal glands, respectively. We performed a retrospective study to assess whether a previously developed normal tissue complication probability (NTCP) model could guide planning to reduce the risk of these complications. We also evaluate if the use of VMAT/IMRT instead of 3D planning can reduce the risk of side effects while maintaining dose to the brain.

**Methods:**

We identified 11 patients who had previously received WBRT to 30 Gy in 10 fractions using 3D‐conformal radiation therapy without prospective delineation of the parotid or lacrimal glands. For each patient, these structures were contoured and new 3D and IMRT plans were created to limit the V_20_ to the parotid glands and the V_15_ to the lacrimal glands while maintaining the dose to the brain. A previously developed relative seriality (RS) NTCP model was used to assess the reduction in xerostomia and dry eye risk relative to the original plan that was achieved with the new plans.

**Results:**

The 3D re‐plans significantly (*p* < 0.001) reduced the estimated risk of xerostomia, by 12.2 ± 4.5%, but did not significantly (*p* > 0.025) reduce the risk of dry eye. The IMRT re‐plans significantly (*p* < 0.001) reduced the risk of xerostomia, by 20.6 ± 7.2%, and dry eye by 11.0 ± 3.8%. Both re‐plans maintained target coverage.

**Conclusion:**

By using parameter values obtained from NTCP models, we were able to create whole brain plans that lowered the estimated risk of xerostomia and dry eye while maintaining target coverage.

## 1 INTRODUCTION

Brain metastases is a potential development in cancer patients, with an estimated incidence of 5%–20%.^1^, ^2^, ^3^ The management of these patients is complex, depending on the size, location, and number of the metastases in the brain, control of the primary disease, and functional status.^4^ For patients who do have brain metastases and are not good candidates for aggressive treatments such as surgery or stereotactic radiosurgery, whole brain radiation (WBRT) is a commonly used palliative treatment.^5^, ^6^, ^7^, ^8^, ^9^ WBRT utilizing two opposed beams has proven to be an effective treatment technique and allows for the avoidance of the lens which are highly radiosensitive.^10^

The prognosis for these patients remains poor, and therefore it is important to minimize treatment related toxicities to avoid significant detriment to the patient's quality of life. Although some of these toxicities, such as neurocognitive effects and fatigue, are difficult or impossible to avoid as they are a result of treating the brain,^11^, ^12^, ^13^ other organs at risk (OARs) can potentially be spared. Past studies have shown that the parotid and lacrimal glands can receive a clinically significant dose during typical whole brain radiotherapy,^14^, ^15^ which can lead to additional side effects for the patient. As such, there has been some work to develop whole brain treatments that allow for increased sparing of these structures.^16^, ^17^ This can be done either by modifying the 3D fields,^16^, ^17^ or by using volume modulated arc therapy (VMAT).^18^

In a recent prospective study, the incidence of clinically significant acute xerostomia and dry eye was reported to be approximately 35% and 25%, respectively.^19^, ^20^, ^21^ In a follow‐up study, the dose delivered to the parotid and lacrimal glands was also analyzed to derive parameter values for two NTCP models, namely the Lyman–Kutcher–Burman (LKB) and relative seriality (RS).^22^ Additionally, dose thresholds were identified, which could aid in treatment plan optimization and assessment.

In this study, we retrospectively re‐planned patients who had previously received WBRT at our institution using 3D and intensity modulated radiation therapy (IMRT) techniques, using these NTCP parameters and dose thresholds to help guide the planning process. Doing this, we evaluated the maximum reasonably achievable risk reduction without compromising target coverage.

## 2 METHODS

### 2.1 Patient selection, treatment, and OAR delineation

From the cohort of our previous studies,^20^, ^22^ eleven patients previously treated with 3D CRT were selected to be re‐planned. Those patients were treated in the supine position using head‐cast immobilization to a total dose of 30 Gy in 10 fractions (3 Gy per fraction). Both the bilateral parotid and lacrimal glands were retrospectively delineated. More specifically, the planning CT was used to contour the parotid glands, and a fused MR was used to contour the lacrimal glands. The initial WBRT fields were designed to cover the skull, and the inferior border was defined by using the C1 or C2 vertebrae.^20^

### 2.2 Treatment planning

**FIGURE 1:**
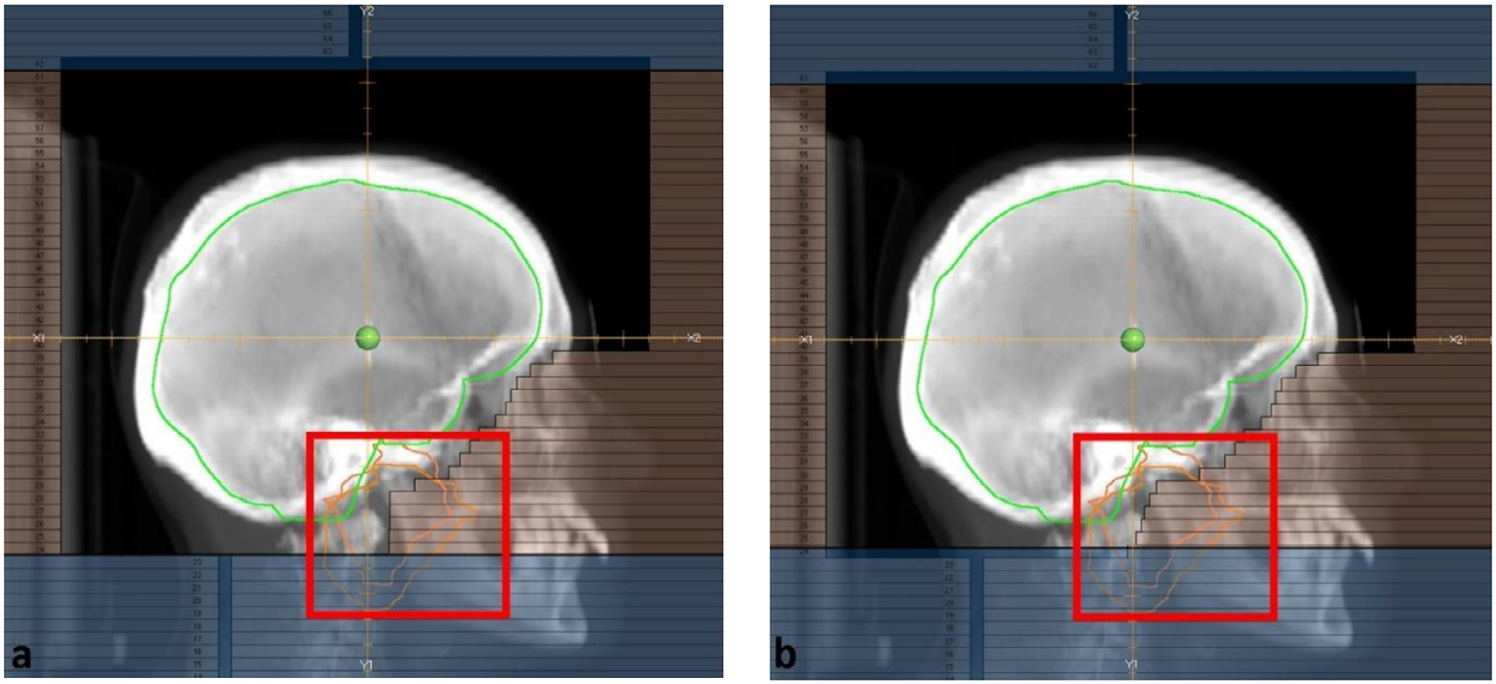
Comparison of the original (a) and re‐planned (b) 3D fields. In (b), the parotid glands (orange) have been further blocked by modifying the field shape in the indicated region. Because of the anatomical location of the lacrimal glands, it was not possible to further spare them in most of the 3D re‐plans.

For the new 3D plans, two parallel‐opposed fields were used (gantry angles: around 85° and 275°, respectively) with beam energy of 6MV. Those fields were adjusted to block the parotid and lacrimal glands, trying however to avoid compromising the coverage of the brain. Figure 1 shows an example of the field adjustment. For the VMAT/IMRT re‐plans, the dose thresholds reported in our earlier study were used to guide optimization to reduce the dose to these structures. For the lacrimal glands, the goal was to minimize the V_15Gy_ metric, with a primary goal to reduce it below 80% and a secondary goal to restrict the max dose to 15 Gy. For the parotid glands, the goal was to minimize the V_20Gy_ metric, with a primary goal of reducing it below 50% and a secondary goal to restrict the max dose to 20 Gy. Per our standard practice for whole brain IMRT, additional optimization objectives were added to reduce the bilateral hippocampal *D*_100%_ ≤ 9 Gy and *D*_max_ ≤ 16 Gy per the NRG‐CC001 hippocampal sparing trial.^23^ An avoidance structure was created for the hippocampi with a 5 mm expansion. Subsequently, per the NRG‐CC001 trial, the planning target volume (PTV) was defined as the whole brain minus the hippocampal avoidance structure. The plans were deemed clinically acceptable if at least 90% of the PTV received the prescription dose. Table 1 shows a summary of the clinical goals used for these plans. Also, the beam settings of the IMRT plan of each patient are shown in Table A1 in the Appendix together with the optimization settings (dose constraints and priority weights) of PTV, parotid and lacrimal glands, respectively.

**TABLE 1:** Summary of the IMRT planning objectives. *D*_x%_ refers to the dose that is received by a specific percentage (x%) of the tissue volume, whereas *V*_xGy_ refers to the volume of the structure that receives a dose of radiation equal to or greater than "x".

Structure	Dosimetric parameter	Goal
PTV	*D*_2%_	≤37.5 Gy
	*D*_98%_	≥25 Gy
	*V*_30Gy_	≥90%, ≥95%^a)^
Bilateral hippocampi	*D*_min_	≤9 Gy
	*D*_max_	≤16 Gy
Bilateral lacrimal glands	*V*_15Gy_	≤80%
	*D*_max_ ^a)^	≤15 Gy
Bilateral parotid glands	*V*_20Gy_	≤50%
	*D*_max_ ^a)^	≤20 Gy

^a)^ Secondary objective

### 2.3 NTCP‐based plan assessment

The dose volume histograms (DVHs) for the bilateral parotid and lacrimal glands were calculated for the original plan, as well as the 3D and IMRT re‐plans. Using those data and the NTCP model parameters shown in Table 2, the level of sparing of the lacrimal and parotid glands was assessed.^22^ As the LKB and RS models were found to have similar goodness of fit, only the RS results are reported here for the sake of brevity. The results were analyzed using a paired *t*‐test. A *p*‐value of < 0.025 indicated a statistically significant difference.

**TABLE 2:** Parameters used for relative seriality (RS) normal tissue complication probability (NTCP) model. *D*_50_ is the dose resulting in a complication rate of 50%, *γ* is the slope (gradient) of the dose response curve and *s* is the parameter that accounts for the volume dependence of the organ.

Structure	Toxicity	*D*_50_ (Gy)	*γ*	*s*
Parotid glands	Xerostomia	24.9	0.26	10^−4^
Lacrimal glands	Dry eye	63.9	0.34	10^−4^

### 2.4 Statistical analysis

We compared the estimated risks of xerostomia and dry eye under the original plans with those under the 3D re‐plans and IMRT re‐plans using paired *t*‐tests. All tests were two‐sided, and for each risk (xerostomia and dry eye), statistical significance was defined as *α* = 0.025 to account for the two comparisons (Bonferroni adjustment). For each patient, the risk difference relative to the original plan was calculated, and the summary statistics (mean ± standard deviation) were reported. Power and sample size calculations were performed in R (version 4.4.0) using the pwr package (version 1.3‐0). Based on clinical judgment, a minimal clinically meaningful change was defined as a 7%–8% reduction in risk, corresponding to a standardized effect size of ∼1.1–1.3, given the observed variability. A standard deviation (SD) of 6.2% was derived from historical data on the estimated risk of xerostomia under the original plan among 11 patients. Therefore, the use of this baseline SD of 6.2% provides a conservative estimate of variability when expressing the standardized effect size (7%–8% ÷ 6.2% ≈ 1.1–1.3). Using an effect size of 1.1, a paired *t*‐test with *n* = 11 provides approximately 82% power at a two‐sided *α* = 0.025.

## 3 RESULTS

**FIGURE 2:**
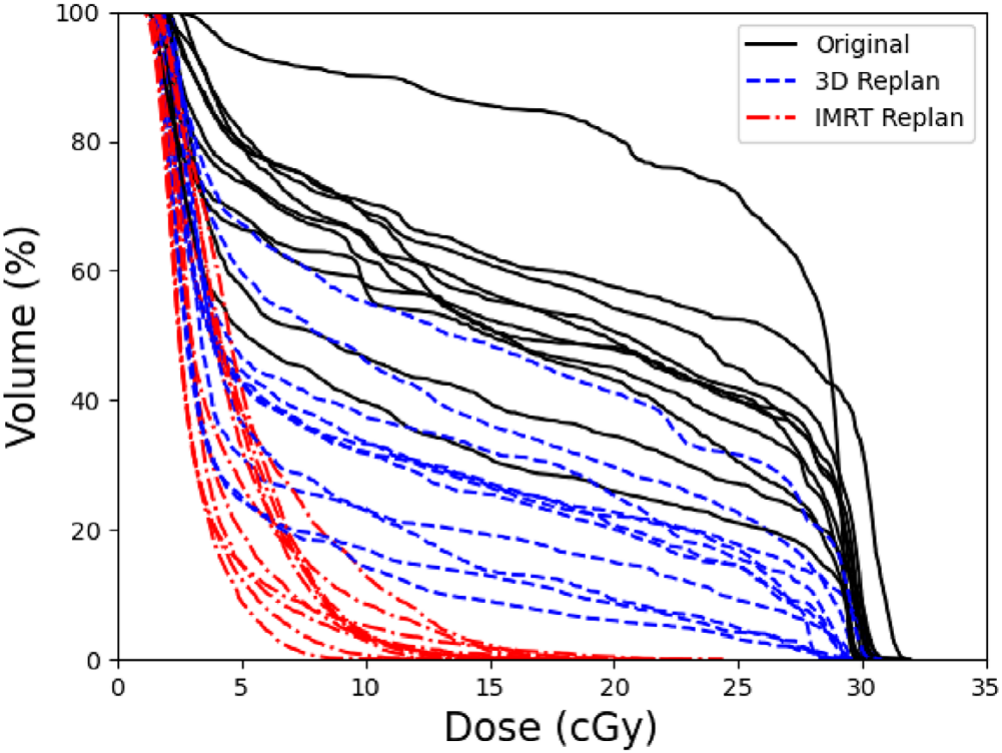
Bilateral parotid dose volume histograms (DVHs) of the original plan and the 3D and IMRT re‐plans for all patients used in this study.

**FIGURE 3:**
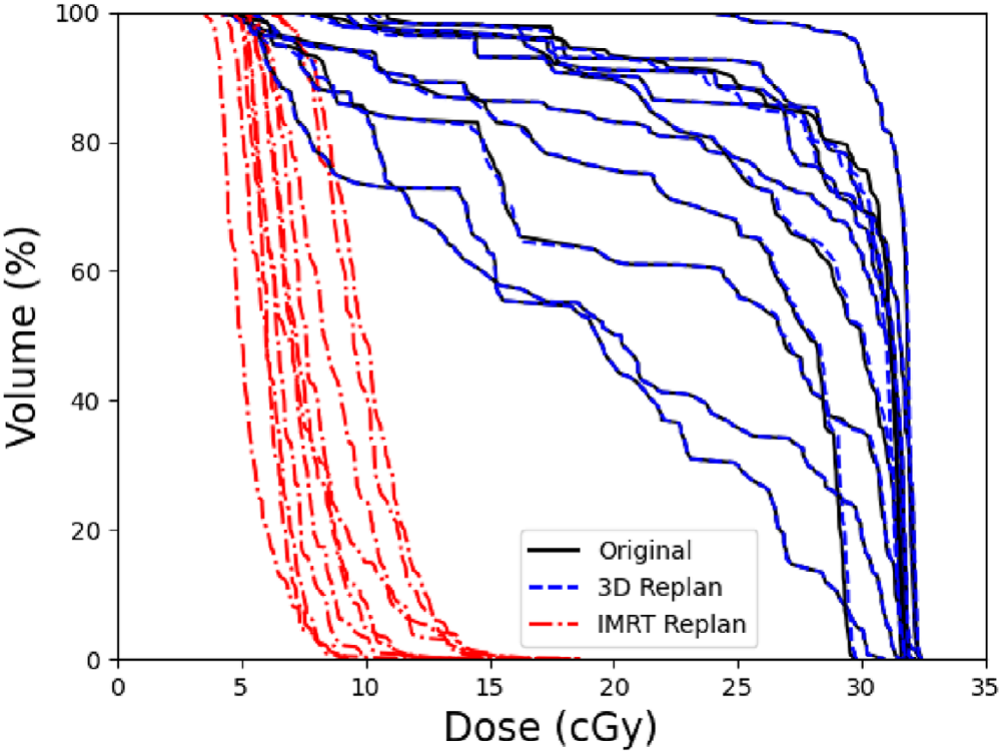
Bilateral lacrimal dose volume histograms (DVHs) of the original plan and the 3D and IMRT re‐plans for all patients used in this study.

**FIGURE 4:**
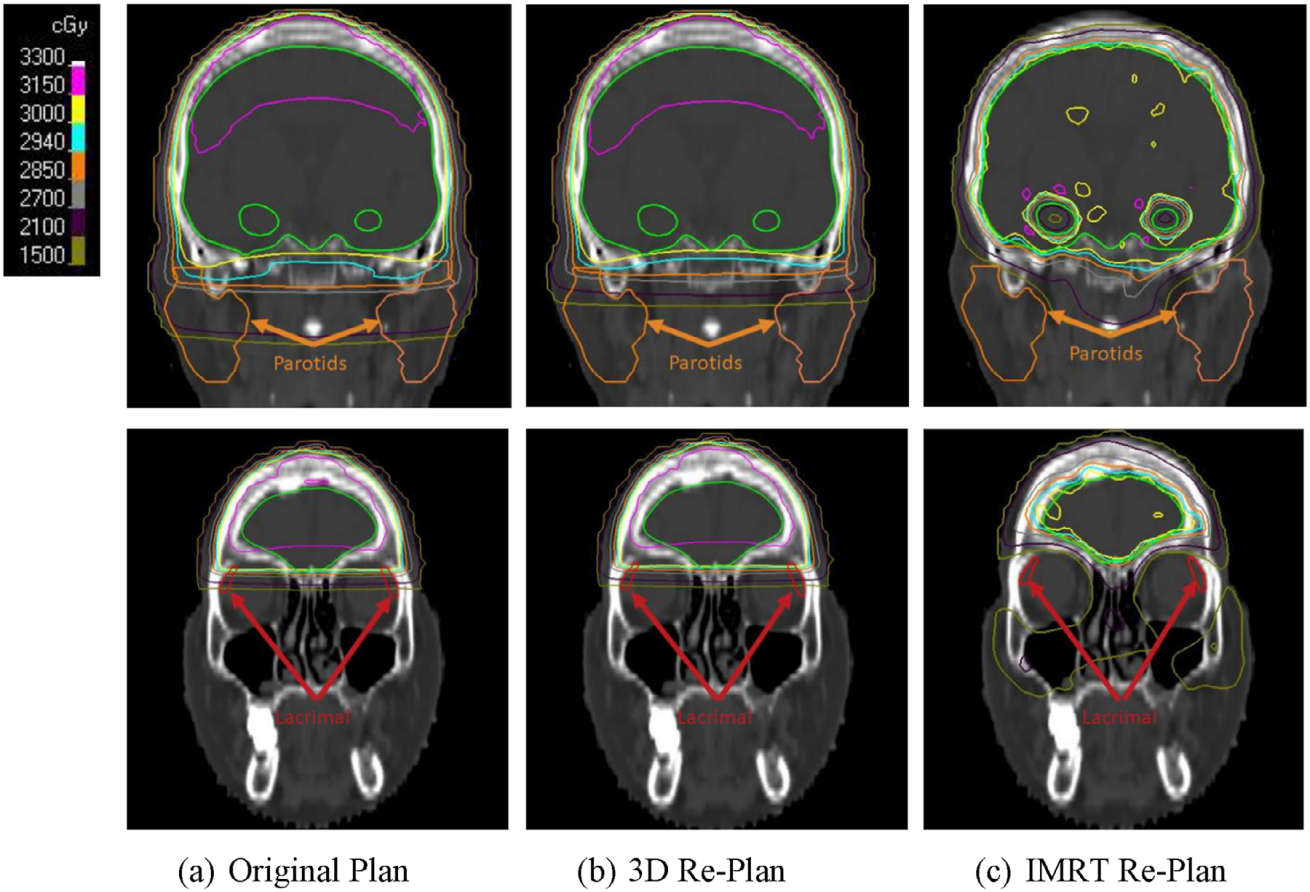
Comparison of the dose distributions of the original WBRT plan (a) and the 3D (b) and IMRT (c) re‐plans focusing on the regions near the parotid glands (top rows) and lacrimal glands (bottom rows).

As shown in Figure 2, both the 3D and IMRT re‐plans were able to reduce dose to the bilateral parotid glands, with the IMRT plans able to achieve greater sparing compared to 3D plans. In the Appendix, Tables A2 and A3 show the comparison of the clinical goals of the 3D and IMRT re‐plans, respectively, to the original plan for each patient. Figure 3 shows that while the IMRT re‐plans were able to better spare the bilateral lacrimal glands compared to the original plans, this was not possible for the 3D replans without compromising coverage of the target. Figure 4 shows the dose distributions of the parotid and lacrimal glands at the coronal planes from the original plan and the re‐plans for a representative patient. This figure visually demonstrates how much the parotid dose could be reduced by the 3D and IMRT re‐plans compared to the original 3D plan, as well as the lever of better sparing that IMRT could achieve regarding the lacrimal glands.

Table 3 shows the comparison of the average calculated NTCP for xerostomia and dry eye between the original plan and the 3D and IMRT re‐plans. The individual NTCP results for all patients included in this study can be found in Tables A4 and A5 in the Appendix. Both the 3D and IMRT re‐plans significantly decreased the calculated risk for xerostomia (*p *< 0.001). Using IMRT significantly decreased the risk compared to the 3D re‐plans (*p *< 0.004). For dry eye, only the IMRT re‐plans were able to significantly decrease the calculated risk (*p *< 0.001).

**TABLE 3:** Comparison of the calculated NTCP results between the different plans.

Toxicity	Plan	NTCP (%)	Δ from original (%)
Xerostomia	Original	33.9 ± 6.2	N/A
	3D re‐plan	21.8 ± 6.3	−12.2 ± 4.5
	IMRT re‐plan	13.4 ± 6.4	−20.6 ± 7.2
Dry eye	Original	22.2 ± 3.5	N/A
	3D re‐plan	21.9 ± 3.4	−0.3 ± 0.9
	IMRT re‐plan	11.2 ± 3.2	−11.0 ± 3.8

Abbreviations: NTCP, normal tissue complication probability.

## 4 DISCUSSION

The dose‐response relations and NTCP model parameters, which were determined by previous studies, were utilized in this study to assess how much clinical benefit may be reasonably achievable by further sparing the bilateral parotid and lacrimal glands during WBRT.

The results of this analysis indicate that it is possible to achieve a significant (*p *< 0.001) reduction in the risk of xerostomia using both 3D and IMRT techniques without a clinically meaningful reduction in dose to the target. The use of IMRT allows an even greater reduction in xerostomia risk, with an average reduction of 20.6% compared to the original 3D plans and 8.4% compared to 3D re‐plans designed to maximize the possible sparing of the parotid glands.

In comparison, only the IMRT re‐plans were able to achieve a significant reduction (*p *< 0.001) in the risk of dry eye, which on average was 11%. Although the use of IMRT allowed for the sparing of the bilateral lacrimal glands, it was generally not possible to block them in the 3D plans due to their proximity to the brain. Though historically 3D, rather than IMRT, has been the modality of choice for WBRT due to the palliative nature of the treatment, recent studies demonstrating the benefit of hippocampal avoidance in WBRT have expanded the use of IMRT or VMAT.^23^, ^24^, ^25^, ^26^ This work further supports the expansion of VMAT/IMRT for WBRT to reduce the risk of dry eye.

In our previous studies,^19^, ^20^, ^21^, ^22^ we have shown that both dry eye and xerostomia are common acute toxicities for patients receiving WBRT. Although several studies have reported the substantial dose received by the parotid and lacrimal glands during treatment,^27^, ^28^, ^29^, ^30^, ^31^, ^32^ in standard practice these organs are not spared, or even routinely delineated. However, as these patients are generally receiving palliative treatment, it is important to consider the impact of those acute effects on the patient's quality of life. This work applies the model parameters that were recently published for the endpoints of xerostomia and dry eye in order to reoptimize deliverable plans and estimate the level of achievable reduction of the risk for those radiation induced toxicities.

One point to clarify is the process of sparing the parotid and lacrimal glands, which are close to PTV since this would be associated with competing objectives and constraints in plan optimization. In all those cases, parotid and lacrimal glands are not overlapping with the PTV. However, parts of them co‐exist with PTV in many axial slices, which resulted in their irradiation in the 3D technique, especially when those OARs were not drawn. Although the 3D technique cannot efficiently spare those OARs in those axial levels, IMRT can do that due to its much larger degrees of freedom. It was shown in this study that the delineation of the lacrimal glands did not improve the capability of the 3D CRT technique to spare them. However, regarding parotid glands, the delineation of those structures was shown to help in sparing some parts of them. Table A1 in the Appendix shows the optimization settings for the parotid and lacrimal glands.

This study includes the following limitations. First, this study involved both the retrospective delineation of the bilateral parotid and lacrimal glands, as well as a retrospective planning for a population that had already undergone treatment. As such, while our NTCP model predicts that the dose reductions achieved in our re‐plans will lower the risk of acute toxicities, we do not yet have clinical data to support this conclusion. Second, although performing this study using a cohort of 11 patients was adequate to provide results that indicate statistical significance for the dosimetric and NTCP differences observed, that size would not be enough for clinical validation. For such a validation a much larger number of cases with complete dosimetric and patient outcome data would be needed to show the real clinical impact of the reported dosimetric differences or NTCP estimations.

## 5 CONCLUSION

The findings of this study indicate that it is possible to create clinically deliverable 3D and IMRT WBRT plans that are predicted to reduce the risk of xerostomia. Additionally, it was shown that the use of IMRT may also lead to a reduction of the risk of dry eye. These findings support that the delineation and sparing of these glands can provide clinical benefit to patients undergoing WBRT, though further clinical study is needed to evaluate the validity of those findings.

## AUTHOR CONTRIBUTIONS

**Gregory Szalkowski**: Conceptualization; data collection / curation; formal data analysis; investigation; methodology; writing the original draft. **Jeffery Fenoli and Mary Oakey**: Conceptualization; data collection/curation; formal data analysis; investigation; methodology; correcting‐original draft. **Xianming Tan**: Performed the statistical analysis. **Kevin A. Pearlstein**: Verified the analytical methods; contributed to the design and implementation of the research; reviewed and corrected the original draft. **Trevor J. Royce and Bhishamjit S. Chera**: Conceptualization; data collection/curation; formal data analysis; investigation; methodology. **Shiva K. Das**: Verified the analytical methods; contributed to the design and implementation of the research; reviewed and corrected the final manuscript. **Kyle Wang**: Verified the analytical methods; contributed to the design and implementation of the research; reviewed and corrected the final manuscript. **Panayiotis Mavroidis**: Supervised the findings of this work; verified the analytical methods; contributed to the design and implementation of the research; reviewed and corrected the final manuscript. All authors discussed the results and contributed to the final manuscript.

## CONFLICT OF INTEREST STATEMENT

The authors declare no conflicts of interest.

## Supporting information



Supporting Information

## References

[1] Davis FG , Dolecek TA , McCarthy BJ , et al. Toward determining the lifetime occurrence of metastatic brain tumors estimated from 2007 United States cancer incidence data. Neuro Oncol. 2012;14:1171‐1177. doi:10.1093/neuonc/nos152 22898372 10.1093/neuonc/nos152 PMC3424213

[2] Schouten LJ , Rutten J , Huveneers HA , et al. Incidence of brain metastases in a cohort of patients with carcinoma of the breast, colon, kidney, and lung and melanoma. Cancer. 2002;94:2698‐2705. doi:10.1002/cncr.10541 12173339 10.1002/cncr.10541

[3] Barnholtz‐Sloan JS , Sloan AE , Davis FG , et al. Incidence proportions of brain metastases in patients diagnosed (1973–2001) in the metropolitan Detroit cancer surveillance system. J Clin Oncol. 2004;22:2865‐2872. doi:10.1200/JCO.2004.12.149 15254054 10.1200/JCO.2004.12.149

[4] Hatiboglu MA , Akdur K , Sawaya R . Neurosurgical management of patients with brain metastasis. Neurosurg Rev. 2020;43:483‐495. doi:10.1007/s10143‐018‐1013‐6 30058049 10.1007/s10143-018-1013-6

[5] Modh A , Burmeister C , Elshaikh MA , et al. Radiation utilization trends in the treatment of brain metastases from non‐small cell lung cancer. Int J Radiat Oncol Biol Phys. 2017;99:E94. doi:10.1016/j.ijrobp.2017.06.815

[6] Trifiletti DM , Sheehan JP , Grover S , et al. National trends in radiotherapy for brain metastases at time of diagnosis of non‐small cell lung cancer. J Clin Neurosci. 2017;45:48‐53. doi:10.1016/j.jocn.2017.08.028 28866073 10.1016/j.jocn.2017.08.028

[7] Rydzewski NR , Khan AJ , Strauss JB , et al. Mortality after stereotactic radiosurgery for brain metastases and implications for optimal utilization: a national cancer database study. Am J Clin Oncol. 2018;41:1142‐1147. doi:10.1097/COC.0000000000000441 29642077 10.1097/COC.0000000000000441

[8] Sandler KA , Shaverdian N , Cook RR , et al. Treatment trends for patients with brain metastases: does practice reflect the data?. Cancer. 2017;123:2274‐2282. doi:10.1002/cncr.30607 28178376 10.1002/cncr.30607

[9] Li J , Brown PD . The diminishing role of whole‐brain radiation therapy in the treatment of brain metastases. JAMA Oncol. 2017;3:1023‐1024. doi:10.1001/jamaoncol.2016.5411 28056128 10.1001/jamaoncol.2016.5411

[10] Henk JM , Whitelocke RA , Warrington AP , et al. Radiation dose to the lens and cataract formation. Int J Radiat Oncol Biol Phys. 1993;25:815‐820. doi:10.1016/0360‐3016(93)90310‐R 8478231 10.1016/0360-3016(93)90310-r

[11] Wong J , Hird A , Zhang L , et al. Symptoms and quality of life in cancer patients with brain metastases following palliative radiotherapy. Int J Radiat Oncol Biol Phys. 2009;75:1125‐1131. doi:10.1016/j.ijrobp.2008.12.013 19231099 10.1016/j.ijrobp.2008.12.013

[12] Caissie A , Nguyen J , Chen E , et al. Quality of life in patients with brain metastases using the EORTC QLQ‐BN20+2 and QLQ‐C15‐PAL. Int J Radiat Oncol Biol Phys. 2012;83:1238‐1245. doi:10.1016/j.ijrobp.2011.09.025 22172909 10.1016/j.ijrobp.2011.09.025

[13] Steinmann D , Paelecke‐Habermann Y , Geinitz H , et al. Prospective evaluation of quality of life effects in patients undergoing palliative radiotherapy for brain metastases. BMC Cancer. 2012;12:283. doi:10.1186/1471‐2407‐12‐283 22780988 10.1186/1471-2407-12-283 PMC3434068

[14] Noh OK , Chun M , Nam SS , et al. Parotid gland as a risk organ in whole brain radiotherapy. Radiother Oncol. 2011;98:223‐226. doi:10.1016/j.radonc.2010.12.013 21300416 10.1016/j.radonc.2010.12.013

[15] Nanda T , Wu CC , Campbell AA , et al. Risk of dry eye syndrome in patients treated with whole‐brain radiotherapy. Med Dosim. 2017;42:357‐362. doi:10.1016/j.meddos.2017.07.007 28784430 10.1016/j.meddos.2017.07.007

[16] Cho O , Chun M , Park SH , et al. Parotid gland sparing effect by computed tomography‐based modified lower field margin in whole brain radiotherapy. Radiat Oncol J. 2013;31:12‐17. doi:10.3857/roj.2013.31.1.12 23620864 10.3857/roj.2013.31.1.12 PMC3633226

[17] Wu CC , Wuu YR , Jani A , et al. Whole‐brain irradiation field design: a comparison of parotid dose. Med Dosim. 2017;42:145‐149. doi:10.1016/j.meddos.2017.02.006 28479012 10.1016/j.meddos.2017.02.006

[18] Sood S , Pokhrel D , McClinton C , et al. Volumetric‐modulated arc therapy (VMAT) for whole brain radiotherapy: not only for hippocampal sparing, but also for reduction of dose to organs at risk. Med Dosim. 2017;42:375‐383. doi:10.1016/j.meddos.2017.07.005 28822604 10.1016/j.meddos.2017.07.005

[19] Wang K , Pearlstein KA , Moon DH , et al. Assessment of risk of xerostomia after whole‐brain radiation therapy and association with parotid dose. JAMA Oncol. 2019;5:221‐228. doi:10.1001/jamaoncol.2018.4951 30489607 10.1001/jamaoncol.2018.4951 PMC6439567

[20] Wang K , Tobillo R , Mavroidis P , et al. Prospective assessment of patient‐reported dry eye syndrome after whole brain radiation. Int J Radiat Oncol Biol Phys. 2019;105:765‐772. doi:10.1016/j.ijrobp.2019.07.015 31351194 10.1016/j.ijrobp.2019.07.015 PMC7384248

[21] Mavroidis P , Pearlstein KA , Moon DH , et al. NTCP modeling of xerostomia related to parotid dose from whole‐brain radiation therapy. Int J Radiat Oncol Biol Phys. 2019;105:E795. doi:10.1016/j.ijrobp.2019.06.2479

[22] Mavroidis P , Pearlstein KA , Moon DH , et al. NTCP modeling and dose–volume correlations for acute xerostomia and dry eye after whole brain radiation. Radiat Oncol. 2021;16:56. doi:10.1186/s13014‐021‐01786‐6 33743773 10.1186/s13014-021-01786-6 PMC7981795

[23] Brown PD , Gondi V , Pugh S , et al. Hippocampal avoidance during whole‐brain radiotherapy plus memantine for patients with brain metastases: phase III trial NRG oncology CC001. J Clin Oncol. 2020;38:1019‐1029. doi:10.1200/JCO.19.02767 32058845 10.1200/JCO.19.02767 PMC7106984

[24] Rong Y , Evans J , Xu‐Welliver M , et al. Dosimetric evaluation of intensity‐modulated radiotherapy, volumetric modulated arc therapy, and helical tomotherapy for hippocampal‐avoidance whole brain radiotherapy. PLOS ONE. 2015;10:e0126222. doi:10.1371/journal.pone.0126222 25894615 10.1371/journal.pone.0126222 PMC4404135

[25] Chia BSH , Leong JY , Ong ALK , et al. Randomised prospective phase II trial in multiple brain metastases comparing outcomes between hippocampal avoidance whole brain radiotherapy with or without simultaneous integrated boost: hA‐SIB‐WBRT study protocol. BMC Cancer. 2020;20:1045‐1049. doi:10.1186/s12885‐020‐07565‐y 33126867 10.1186/s12885-020-07565-y PMC7602352

[26] Weiner JP . Neurocognitive outcomes for patients with brain metastasis in the modern era: benefit of treatment with hippocampal avoidance whole‐brain radiotherapy plus memantine. J Clin Oncol. 2020;38:1003‐1005. doi:10.1200/JCO.19.03359 32058847 10.1200/JCO.19.03359

[27] Nanda T , Wu CC , Campbell AA , et al. Risk of dry eye syndrome in patients treated with whole‐brain radiotherapy. Med Dosim. 2017;42:357‐362. doi:10.1016/j.meddos.2017.07.007 28784430 10.1016/j.meddos.2017.07.007

[28] Avkshtol V , Johnson ME , Ruth KJ , et al. Lacrimal gland radiation dose and toxicity after whole‐brain radiation [abstract]. Int J Radiat Oncol Biol Phys. 2014;90:S701. doi:10.1016/j.ijrobp.2014.05.2055

[29] Wu CC , Wuu YR , Jani A , et al. Whole‐brain irradiation field design: a comparison of parotid dose. Med Dosim. 2017;42:145‐149. doi:10.1016/j.meddos.2017.02.006 28479012 10.1016/j.meddos.2017.02.006

[30] Cho O , Chun M , Park SH , et al. Parotid gland sparing effect by computed tomography‐based modified lower field margin in whole brain radiotherapy. Radiat Oncol J. 2013;31:12‐17. doi:10.3857/roj.2013.31.1.12 23620864 10.3857/roj.2013.31.1.12 PMC3633226

[31] Noh OK , Chun M , Nam SS , et al. Parotid gland as a risk organ in whole brain radiotherapy. Radiother Oncol. 2011;98:223‐226. doi:10.1016/j.radonc.2010.12.013 21300416 10.1016/j.radonc.2010.12.013

[32] Orton A , Gordon J , Vigh T , et al. Differences in parotid dosimetry and expected normal tissue complication probabilities in whole brain radiation plans covering C1 versus C2. Cureus. 2017;9:e1217. 28589066 10.7759/cureus.1217 PMC5453748

