# Supplementary material for: NTCP model guided whole brain radiation re‐planning to reduce risk of acute xerostomia and dry eye
Source: J Appl Clin Med Phys. 2025 Nov 18;26(12):e70344. doi: 10.1002/acm2.70344 (PMC12626740; doi:10.1002/acm2.70344)
Supplement: Supplementary file 1 — Supporting Information [file ACM2-26-e70344-s001.docx]

**APPENDIX**

**TABLE A1** Summary of the VMAT/IMRT plan configurations of each patient. The optimization dose constraints and priority weights of the parotid and lacrimal glands are also shown. In all the cases, the beams energy was 6MV and the leaf motion constraint was 0.48 cm/deg.

| **Patients** | **Beam Settings** | | | | **Optimization Settings** | | | |
| --- | --- | --- | --- | --- | --- | --- | --- | --- |
| **Patient 1** | **Parameters** | **Beam 1** | **Beam 2** | **Beam 3** | **Structure** | **Lacrimal** | **Parotid** | **PTV** |
|  | Gantry angles | 179-181 CCW | 181-179 CW | 181-45 CW | Dose constraints | V_12_ < 30% | V_8.5_ < 20% | V_30_ < 99% |
|  | Collimator angles | 330 | 30 | 10 | Priority weights | 20 | 20 | 400 |
|  | Couch rotation | 0 | 0 | 90 | Dose constraints | V_15_ < 10% | D_max_ < 20Gy | Uniform 30Gy |
|  | No of segments | 180 | 180 | 113 | Priority weights | 10 | 10 | 100 |
| **Patients 2, 3, 4, 5, 6** | Gantry angles | 179-181 CCW | 181-179 CW | 181-45 CW | Dose constraints  Priority weights | V_12_ < 30% | V_8.5_ < 20% | V_30_ < 99% |
|  | Collimator angles | 330 | 30 | 45 |  | 20 | 20 | 400 |
|  | Couch rotation | 0 | 0 | 90 | Dose constraints | V_15_ < 10% | D_max_ < 20Gy | Uniform 30Gy |
|  | No of segments | 180 | 121 | 113 | Priority weights | 10 | 10 | 100 |
| **Patients 7, 8, 9, 10, 11** | Gantry angles | 179-181 CCW | 181-179 CW | 181-0 CW | Dose constraints  Priority weights | V_12_ < 30% | V_8.5_ < 20% | V_30_ < 99% |
|  | Collimator angles | 330 | 30 | 90 |  | 20 | 20 | 400 |
|  | Couch rotation | 0 | 0 | 90 | Dose constraints | V_15_ < 10% | D_max_ < 20Gy | Uniform 30Gy |
|  | No of segments | 180 | 121 | 91 | Priority weights | 10 | 10 | 100 |

**TABLE A2** Comparison of the clinical goals for the 3D re-plan vs. the original plan for each patient included in this study (the units are expressed in Gy unless otherwise noted).

| **ROI** | **Clinical Goal** | **Pt 1** | **Pt 2** | **Pt 3** | **Pt 4** | **Pt 5** | **Pt 6** | **Pt 7** | **Pt 8** | **Pt 9** | **Pt 10** | **Pt 11** | **Average** |
| --- | --- | --- | --- | --- | --- | --- | --- | --- | --- | --- | --- | --- | --- |
| PTV | At least 90% volume at 30 Gy dose | -2.2% | -1.4% | -1.5% | -0.3% | -0.7% | -0.7% | -1.9% | -1.9% | -0.6% | -2.0% | -1.4% | -1.3% |
|  | At least 25 Gy dose at 98% volume | -0.3 | 0.1 | -0.2 | -0.1 | -0.1 | -0.5 | -0.5 | -0.2 | -0.1 | -0.2 | -0.2 | -0.2 |
|  | At most 37.5 Gy dose at 2% volume | 0.1 | 0.0 | 0.1 | 0.0 | 0.1 | 0.1 | 0.1 | 0.0 | 0.0 | 0.1 | 0.0 | 0.0 |
| Hippocampus Lt | At most 9 Gy dose at 100% volume | -0.1 | 0.0 | 0.0 | -0.1 | 0.0 | -0.2 | -0.1 | -0.1 | 0.0 | -0.1 | 0.0 | -0.1 |
|  | At most 16 Gy dose at 0.10 cc volume | -0.1 | 0.0 | -0.1 | -0.1 | -0.1 | 0.0 | -0.1 | -0.1 | 0.0 | 0.0 | -0.1 | -0.1 |
| Hippocampus Rt | At most 9 Gy dose at 100% volume | 0.0 | 0.0 | 0.0 | -0.1 | 0.0 | -0.2 | -0.1 | -0.1 | 0.0 | -0.1 | 0.0 | -0.1 |
|  | At most 16 Gy dose at 0.10 cc volume | -0.1 | -0.2 | -0.1 | -0.1 | 0.0 | 0.0 | 0.0 | -0.1 | 0.0 | 0.0 | -0.1 | -0.1 |
| Brainstem | At most 33 Gy dose 0.10 cc volume | -0.2 | 0.0 | -0.3 | -0.2 | -0.4 | -0.4 | -0.3 | -0.1 | 0.1 | -0.3 | -0.1 | -0.2 |
| Chiasm | At most 33 Gy dose 0.10 cc volume | -0.1 | 0.0 | -0.1 | -0.1 | 0.0 | -0.2 | -1.6 | 0.0 | 0.0 | -0.1 | 0.0 | -0.2 |
| Optic Nerve Lt | At most 33 Gy dose 0.10 cc volume | 0.0 | 0.0 | 0.0 | -0.1 | -0.3 | -0.4 | 0.1 | 0.0 | 0.0 | 0.0 | 0.0 | 0.0 |
| Optic Nerve Rt | At most 33 Gy dose 0.10 cc volume | 0.0 | 0.1 | -0.1 | 0.0 | -0.1 | -0.4 | 0.0 | 0.0 | 0.0 | 0.0 | 0.0 | 0.0 |
| Globe Lt | At most 10 Gy dose at 0.10 cc volume | 0.1 | 0.1 | 0.0 | 0.0 | 0.1 | -0.2 | 0.1 | 0.0 | 0.0 | 0.0 | 0.0 | 0.0 |
| Globe Rt | At most 10 Gy dose at 0.10 cc volume | 0.2 | -0.1 | 0.0 | 0.0 | 0.0 | -0.3 | 0.2 | 0.0 | 0.0 | 0.0 | 0.0 | 0.0 |
| Lens Lt | At most 5 Gy dose at 0.10 cc volume | -0.1 | -0.1 | 0.0 | 0.0 | 27.8 | 0.2 | 0.0 | 0.0 | 0.0 | -0.1 | 0.0 | 2.5 |
| Lens Rt | At most 5 Gy dose at 0.10 cc volume | 0.0 | 0.0 | -0.1 | 0.0 | 0.0 | 0.3 | -0.1 | 0.0 | 0.0 | -0.1 | 0.0 | 0.0 |
| Lacrimal Lt | At most 80% volume at 15 Gy dose | 0.0% | 0.0% | 0.0% | 0.0% | -3.1% | 0.0% | 0.7% | -0.1% | 0.0% | 0.0% | 0.0% | -0.2% |
| Lacrimal Rt | At most 80% volume at 15 Gy dose | 0.0% | 0.0% | 0.0% | -0.1% | 0.0% | 0.0% | -0.9% | 0.0% | 0.0% | 0.0% | 0.0% | -0.1% |
| Parotid Lt | At most 50% volume at 20 Gy dose | -26.5% | -33.2% | -32.3% | -21.3% | -16.0% | -40.0% | -53.3% | -34.8% | -13.0% | -27.1% | -18.6% | -28.7% |
| Parotid Rt | At most 50% volume at 20 Gy dose | -23.9% | -28.2% | -44.7% | -17.8% | -23.3% | -32.6% | -57.4% | -32.9% | -13.2% | -28.0% | -20.1% | -29.3% |
| Optics+3mm | At most 37.5 Gy dose at 0.03 cc volume | 0.0 | 0.1 | -0.1 | 0.0 | -0.1 | -0.4 | 0.0 | 0.0 | 0.0 | 0.0 | 0.0 | 0.0 |
|  | At most 30 Gy dose at 0.03 cc volume | 0.0 | -0.1 | -0.1 | 0.0 | -0.1 | -0.4 | 0.0 | 0.0 | 0.0 | 0.0 | 0.0 | -0.1 |

**TABLE A3** Comparison of the clinical goals for the IMRT re-plan vs. the original plan for each patient included in this study (the units are expressed in Gy unless otherwise noted).

| **ROI** | **Clinical Goal** | **Pt 1** | **Pt 2** | **Pt 3** | **Pt 4** | **Pt 5** | **Pt 6** | **Pt 7** | **Pt 8** | **Pt 9** | **Pt 10** | **Pt 11** | **Average** |
| --- | --- | --- | --- | --- | --- | --- | --- | --- | --- | --- | --- | --- | --- |
| PTV | At least 90% volume at 30 Gy dose | -4.0% | -5.4% | -4.1% | -6.4% | -7.7% | 12.2% | 6.3% | -5.9% | -6.0% | -4.7% | -4.7% | -2.8% |
|  | At least 25 Gy dose at 98% volume | -0.5 | -0.9 | -0.5 | -1.1 | -1.2 | -0.1 | -0.9 | -1.3 | -0.7 | -0.8 | -0.8 | -0.8 |
|  | At most 37.5 Gy dose at 2% volume | -0.9 | -1.0 | -1.3 | -0.6 | -0.8 | -0.3 | 0.4 | -0.6 | -1.2 | -0.7 | -0.7 | -0.7 |
| Hippocampus Lt | At most 9 Gy dose at 100% volume | -21.2 | -21.0 | -21.0 | -21.6 | -21.2 | -21.1 | -21.1 | -21.4 | -21.3 | -21.5 | -21.5 | -21.3 |
|  | At most 16 Gy dose at 0.10 cc volume | -16.6 | -14.6 | -15.2 | -16.1 | -15.9 | -14.5 | -14.4 | -15.2 | -15.7 | -15.8 | -15.8 | -15.4 |
| Hippocampus Rt | At most 9 Gy dose at 100% volume | -21.1 | -20.9 | -20.8 | -21.8 | -21.3 | -20.9 | -20.7 | -21.2 | -20.9 | -21.1 | -21.1 | -21.1 |
|  | At most 16 Gy dose at 0.10 cc volume | -17.5 | -15.0 | -15.8 | -16.0 | -16.1 | -14.1 | -15.0 | -15.7 | -15.7 | -15.9 | -15.9 | -15.7 |
| Brainstem | At most 33 Gy dose 0.10 cc volume | 1.4 | 1.4 | 1.4 | 0.7 | 1.0 | 2.6 | 3.3 | 2.0 | 0.8 | 2.0 | 2.0 | 1.7 |
| Chiasm | At most 33 Gy dose 0.10 cc volume | 0.5 | 0.6 | 0.4 | 0.3 | 0.2 | 1.1 | 0.0 | 0.9 | 0.6 | -0.6 | -0.6 | 0.3 |
| Optic Nerve Lt | At most 33 Gy dose 0.10 cc volume | 0.0 | -3.1 | -2.3 | -0.6 | -4.1 | -6.0 | -2.0 | -1.6 | -0.6 | -1.5 | -1.5 | -2.1 |
| Optic Nerve Rt | At most 33 Gy dose 0.10 cc volume | -0.3 | -1.4 | -2.8 | -0.5 | -1.2 | -5.8 | 1.6 | -2.3 | -2.2 | -0.6 | -0.6 | -1.5 |
| Globe Lt | At most 10 Gy dose at 0.10 cc volume | -21.7 | -21.6 | -23.1 | -20.2 | -21.6 | -22.0 | -20.1 | -20.4 | -23.2 | -23.0 | -23.0 | -21.8 |
| Globe Rt | At most 10 Gy dose at 0.10 cc volume | -21.7 | -22.8 | -22.8 | -20.8 | -21.9 | -21.5 | -18.7 | -21.2 | -23.2 | -23.2 | -23.2 | -21.9 |
| Lens Lt | At most 5 Gy dose at 0.10 cc volume | 0.4 | -0.9 | -3.4 | -0.8 | 0.2 | -0.6 | -0.4 | 0.5 | -0.6 | -3.3 | -3.3 | -1.1 |
| Lens Rt | At most 5 Gy dose at 0.10 cc volume | -1.3 | -0.1 | -4.9 | -0.3 | -0.2 | 1.5 | -0.3 | 0.5 | 0.0 | -3.7 | -3.7 | -1.1 |
| Lacrimal Lt | At most 80% volume at 15 Gy dose | -94.5% | -100% | -88.1% | -63.5% | -66.5% | -99.4% | -98.0% | -55.7% | -97.2% | -100% | -100% | -87.5% |
| Lacrimal Rt | At most 80% volume at 15 Gy dose | -99.9% | -96.6% | -100% | -62.7% | -88.4% | -93.4% | -73.9% | -63.3% | -94.9% | -100% | -100% | -88.5% |
| Parotid Lt | At most 50% volume at 20 Gy dose | -45.2% | -54.1% | -47.0% | -50.1% | -18.0% | -52.2% | -81.8% | -47.6% | -56.8% | -35.3% | -35.3% | -47.6% |
| Parotid Rt | At most 50% volume at 20 Gy dose | -50.3% | -47.6% | -87.3% | -48.3% | -31.6% | -37.7% | -79.5% | -38.6% | -51.5% | -33.8% | -33.8% | -49.1% |
| Optics+3mm | At most 37.5 Gy dose at 0.03 cc volume | 0.0 | -0.4 | -0.8 | 0.9 | -0.4 | 0.4 | 1.8 | 0.7 | -0.1 | -0.1 | -0.1 | 0.2 |
|  | At most 30 Gy dose at 0.03 cc volume | 0.0 | -0.4 | -0.8 | 0.9 | -0.4 | 0.4 | 1.8 | 0.7 | -0.1 | -0.1 | -0.1 | 0.2 |

**TABLE A4** Summary of the normal tissue complication probability (NTCP) and biologically effective uniform dose (BEUD) of the parotid glands for the endpoint of xerostomia for each patient included in this study.

| **Pt** | **Parotid glands NTCP (%)** | | | **Parotid glands BEUD (Gy)** | | |
| --- | --- | --- | --- | --- | --- | --- |
|  | Original | 3D Replan | IMRT Replan | Original | 3D Replan | IMRT Replan |
| 1 | 34.96 | 23.97 | 17.23 | 15.24 | 8.13 | 3.30 |
| 2 | 36.10 | 23.10 | 0.90 | 15.96 | 7.53 | 0.10 |
| 3 | 24.35 | 7.38 | 15.73 | 8.38 | 0.10 | 2.12 |
| 4 | 36.01 | 27.75 | 16.58 | 15.91 | 10.64 | 2.80 |
| 5 | 26.27 | 17.87 | 0.01 | 9.66 | 3.79 | 0.10 |
| 6 | 32.97 | 18.57 | 15.25 | 13.98 | 4.31 | 1.74 |
| 7 | 47.29 | 24.74 | 16.77 | 23.11 | 8.65 | 2.95 |
| 8 | 33.42 | 19.86 | 15.13 | 14.27 | 5.25 | 1.65 |
| 9 | 38.10 | 31.93 | 16.59 | 17.22 | 13.33 | 2.81 |
| 10 | 34.57 | 23.38 | 16.46 | 15.00 | 7.72 | 2.71 |
| 11 | 29.40 | 20.93 | 16.58 | 11.71 | 6.02 | 2.80 |

**TABLE A5** Summary of the normal tissue complication probability (NTCP) and biologically effective uniform dose (BEUD) of the lacrimal glands for the endpoint of dry eye for each patient included in this study.

| **Pt** | **Lacrimal glands NTCP (%)** | | | **Lacrimal glands BEUD (Gy)** | | |
| --- | --- | --- | --- | --- | --- | --- |
|  | Original | 3D Replan | IMRT Replan | Original | 3D Replan | IMRT Replan |
| 1 | 23.04 | 23.12 | 10.97 | 26.87 | 26.87 | 6.49 |
| 2 | 24.78 | 24.81 | 20.58 | 29.31 | 29.31 | 23.09 |
| 3 | 24.68 | 21.63 | 9.63 | 29.13 | 24.67 | 3.66 |
| 4 | 16.15 | 16.14 | 10.21 | 16.01 | 16.01 | 4.92 |
| 5 | 19.40 | 19.39 | 10.70 | 21.28 | 21.25 | 5.93 |
| 6 | 24.80 | 24.46 | 10.10 | 29.29 | 28.81 | 4.68 |
| 7 | 20.13 | 20.21 | 10.00 | 22.40 | 22.51 | 4.46 |
| 8 | 16.75 | 16.75 | 9.38 | 17.02 | 17.01 | 3.10 |
| 9 | 24.48 | 24.49 | 10.13 | 28.84 | 28.85 | 4.75 |
| 10 | 22.92 | 22.92 | 11.10 | 26.58 | 26.58 | 6.76 |
| 11 | 26.84 | 26.87 | 10.55 | 32.19 | 32.23 | 5.63 |
